# Synthesis of Functional Materials Using N‐heterocyclic Amines Beyond Melamine

**DOI:** 10.1002/advs.202510137

**Published:** 2025-09-12

**Authors:** Jesús Barrio, Arne Thomas

**Affiliations:** ^1^ Department of Chemical Engineering Imperial College London London SW7 2AZ UK; ^2^ Institute for Chemistry Division of Functional Materials Technische Universität Berlin 10623 Berlin Germany

**Keywords:** carbon materials, covalent organic polymers, electrocatalysis, heterocyclic amines, porous materials

## Abstract

Melamine, a nitrogen‐rich heterocycle with a 1,3,5‐triazine core and three exocyclic amines, has played a central role in materials chemistry, enabling the synthesis of supramolecular assemblies, covalent organic polymers, and carbon nitride semiconductors. However, structurally related N‐heterocyclic amines, differing in ring substitution or heterocycle, are far less applied in such fields despite their potential to overcome limitations associated with melamine‐based materials. In this review, recent advances in the synthesis of covalent and carbon‐based materials derived from melamine analogues, including 6‐Phenyl‐1,3,5‐triazine‐2,4‐diamine, 6‐Methyl‐1,3,5‐triazine‐2,4‐diamine and 2,4,6‐Triaminopyrimidine are highlighted. It is focused on how variations in heterocycle identity and functional group substitution influence monomer reactivity, condensation behavior, and the resulting material properties, such as chemical composition, dimensionality, porosity and morphology. Diverse applications of such materials are discussed, from fluorescent sensing platforms to CO_2_ capture and photo‐electrocatalysis, however, the primary emphasis lies on synthetic strategies and structure‐property relationships. It is concluded with a critical perspective on how these alternative building blocks, when leveraging sustainable synthesis methods coupled with emerging AI‐guided materials discovery, may enable the targeted design of next‐generation functional materials.

## Graphical abstract

Melamine has long enabled supramolecular assemblies, polymers, and carbon nitrides, yet related N‐heterocyclic amines remain underexplored. This review highlights advances with melamine analogues such as 6‐phenyl‐, 6‐methyl‐triazine diamines and 2,4,6‐triaminopyrimidine, focusing on how structural variation governs reactivity, condensation, and material properties, with implications for catalysis, sensing, and CO_2_ capture amongst others.

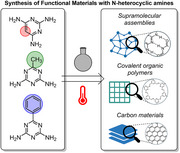

## 1 Melamine as a Building Block for Materials Design

Carbon and nitrogen‐based covalent materials have a crucial role in fields such as energy conversion, photochemistry or sensing.^[^ ^1^ ^]^ The introduction of nitrogen functionalities in a carbon backbone entails the charge redistribution around the neighbor carbon atoms,^[^ ^2^, ^3^ ^]^ and the increase in electronic conductivity due to the increased carrier density. However, in higher concentrations results in semiconducting behavior, as increased localized electronic states hinder charge transport.^[^ ^4^, ^5^, ^6^ ^]^ Graphene‐like sp^2^ carbon frameworks with nitrogen functionalities (usually between 1–10 wt.%) have been widely studied as electrocatalysts,^[^ ^7^, ^8^ ^]^ owing to their high conductivity and reactivity, while porous carbon‐based polymers (sp^3^‐sp^2^) with nitrogen doping have been widely applied as photo‐electrocatalysts, in organic electronics and overall, in a wide variety of energy applications owing to their electronic structure and redox properties,^[^ ^9^, ^10^, ^11^, ^12^, ^13^ ^]^ as well as the tunability of the synthetic strategies.^[^ ^14^ ^]^ To synthesize such functional materials, often bottom‐up approaches are employed which entail the reaction between co‐monomers to form covalent porous networks, or the self‐condensation of C─N based building blocks into polymeric units.^[^ ^15^ ^]^

**Figure 1:**
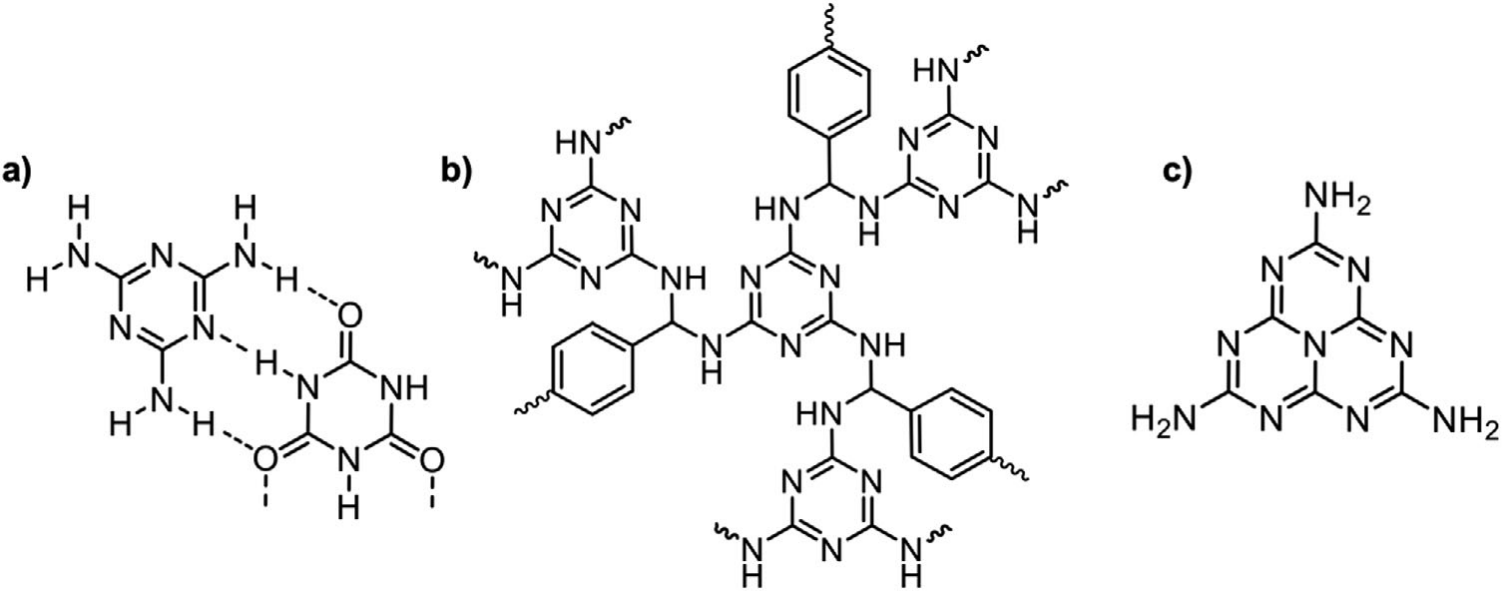
Representation of the hydrogen bonding interactions of melamine with cyanuric acid a),^[^ ^84^ ^]^ the structure of a Schiff‐base polymer with terephthalaldehyde b),^[^ ^44^ ^]^ and the structure of melem.^[^ ^63^ ^]^

In this regard, melamine (1,3,5‐triazine‐2,4,6‐triamine), has emerged as one of the most researched building blocks for the design of covalent materials and the doping of carbon frameworks with nitrogen functionalities.^[^ ^16^, ^17^, ^18^ ^]^ Melamine displays supramolecular functionality (**Figure** **1**a) owing to its three amine groups and three sp^2^ nitrogen within its heterocycle which has been leveraged in the field of supramolecular hydrogels,^[^ ^19^, ^20^ ^]^ composites,^[^ ^21^ ^]^ liquid crystals^[^ ^22^, ^23^ ^]^ or membranes amongst others.^[^ ^24^, ^25^, ^26^, ^27^, ^28^ ^]^ The structures obtained by self‐assembly can serve as precursors to C─N/based semiconductors through controlled thermal conversion.^[^ ^29^, ^30^, ^31^, ^32^, ^33^ ^]^ Besides hydrogen‐bonded self‐assemblies, melamine can form coordination structures,^[^ ^34^, ^35^, ^36^, ^37^, ^38^ ^]^ often in the presence of hydrochloric acid, which protonates the sp^2^ heterocyclic nitrogen resulting in 3D assemblies driven also by *π*–*π* stacking.^[^ ^39^ ^]^

Additionally, melamine can form microporous polymers via Schiff‐base condensation reaction in the presence of aldehydes (Figure 1b); and while the reaction between the ‐NH_2_ groups of melamine and aldehydes has been exploited in the field of melamine‐formaldehyde resins, these are formed via hydroxymethylation and condensation, rather than Schiff‐base, leading to cross‐linked polymers.^[^ ^40^, ^41^ ^]^ The Schiff‐base condensation entails the reaction between an amine and an aldehyde to form an imine linkage,^[^ ^42^ ^]^ and in the case that the amine group is strongly nucleophilic (due strong electron‐donating groups in the vicinity), a secondary nucleophilic attack to the imine carbon would form an aminal bond. This mechanism is particularly relevant in melamine‐based Schiff‐base polymers, where aminal bonds dominate due to the reactivity of melamine's multiple amine groups as well as its symmetry.^[^ ^43^ ^]^ Since the seminal report by Schwab et al.,^[^ ^44^ ^]^ such porous organic polymers have been widely explored in photocatalysis (owing to their semiconducting behavior),^[^ ^45^, ^46^ ^]^ gas sorption and water cleaning (due to their intrinsic porosity),^[^ ^47^, ^48^, ^49^, ^50^, ^51^, ^52^, ^53^, ^54^ ^]^ as templates to porous carbons^[^ ^55^, ^56^, ^57^, ^58^, ^59^ ^]^ as catalyst support,^[^ ^60^, ^61^ ^]^ and even as electrocatalysts.^[^ ^62^ ^]^

However, there is no doubt that melamine has gained the most attention owing to the product of its self‐condensation; graphitic carbon nitride,^[^ ^6^ ^]^ a 2.7 eV semiconductor which is composed of heptazine sub‐units resulting from melamine trimerization, melem (2,5,8‐triamino‐heptazine, Figure 1c).^[^ ^63^ ^]^ Melamine, however, sublimes during thermal condensation allowing the chemical vapor deposition synthesis of films,^[^ ^64^ ^]^ but also leading to relatively low yields, reason why other C─N based precursors such as urea or supramolecular assemblies are used as precursors.^[^ ^31^, ^65^ ^]^ Schnick and co‐workers proposed that the formation of melem is a stepwise condensation resulting from the nucleophilic attack of cyanamide residues that arise from melamine decomposition.^[^ ^66^ ^]^ Melem subunits can then react to form the fully condensed version of carbon nitride; “melon”.^[^ ^67^ ^]^ Albeit a hydrogen bonded complex of less condensed melem subunits has also been proposed as the most feasible structure of polymeric carbon nitride.^[^ ^68^ ^]^ Carbon nitride polymers are an extremely versatile platform for photo‐catalysis, photo‐electrochemistry and heterogeneous catalysis. Recent progress in the synthesis and application of these materials has been extensively reviewed elsewhere and will not be discussed here. Readers are encouraged to refer to those articles.^[^ ^69^, ^70^, ^71^, ^72^, ^73^, ^74^, ^75^, ^76^ ^]^

Semiconductive carbon nitride polymers, however, are stable usually up until 700 °C, which means that melamine by itself is often not a suitable building block to construct conductive carbon‐based materials via high‐temperature pyrolysis. Nevertheless, it can be used as doping agent along with a carbon‐rich building block (such as a polymer or a carbon allotrope)^[^ ^77^, ^78^ ^]^ to insert nitrogen functionalities that provide electrochemical reactivity as well as coordination sites to anchor metallic atoms.^[^ ^79^, ^80^, ^81^, ^82^, ^83^ ^]^

### 1.1 Available Melamine Analogues

**Figure 2:**
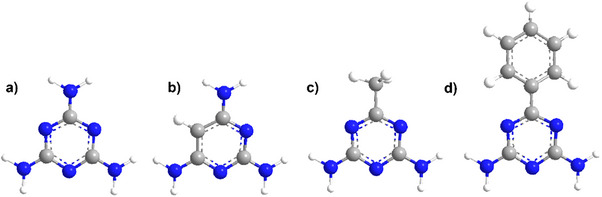
Chemical structure of melamine a), TAP b), AGA c), and BGA d). Nitrogen atoms are represented in blue, carbon atoms in grey and hydrogen atoms in white.

Beyond melamine, the family of N‐heterocyclic amines expands to building blocks like as 2,4,6‐Triaminopyrimidine (TAP), acetoguanamine (6‐Methyl‐1,3,5‐triazine‐2,4‐diamine, AGA), or benzoguanamine (6‐Phenyl‐1,3,5‐triazine‐2,4‐diamine, BGA). Such organic molecules share similar core chemical structures to melamine with mild modifications (**Figure** **2**), and they still provide supramolecular reactivity as well as the possibility to design covalent and carbon‐based functional materials via covalent chemistry and self‐condensation. The structural differences, however, lead to dramatic differences in reactivity and in the properties of the final materials. All the above‐mentioned analogues display a higher C/N ratio than melamine (in weight, 0.428 for melamine, 0.686 for both TAP and AGA and 1.54 for BGA), which suggest the possibility of synthesizing condensates with higher conductivity compared to melamine. AGA and BGA maintain the triazine core, but one amine group is substituted with a methyl (‐CH_3_) and phenyl group (‐C_6_H_5_), respectively, which impact the supramolecular functionality by decreasing the number of hydrogen‐bonding donors and leads to steric effects which impact the dimensionality of the assemblies. Additionally, their electron withdrawing nature (compared to ‐NH_2_ groups in melamine) results in a weakened electron density in the triazine ring, reducing the bond energy of ─C═N─ and therefore requiring lower temperatures to achieve ring‐opening and polymerization reactions.^[^ ^85^ ^]^ TAP on the other hand maintains the three amino group substituents (‐NH_2_) albeit the heterocycle is based on pyrimidine; a 1,3,5‐triazine ring with a C‐H replacing one of the N.

**Figure 3:**
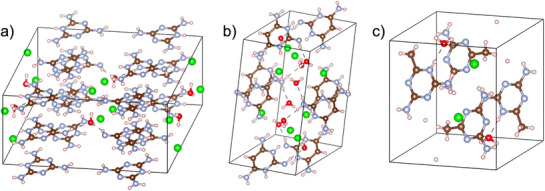
Packing structure of melaminium chloride hemihydrate a), 2,4,6‐triamino‐pyrimidinediium dichloride dihydrate b) and acetoguanaminium chloride c). Hydrogen atoms are represented in pink, nitrogen atoms in blue, carbon atoms in brown, oxygen atoms in red and chlorine atoms in green. Structural visualization was carried out using the VESTA software.^[^ ^97^ ^]^

In the case of supramolecular assemblies created via protonation with hydrochloric acid, melamine forms a 3D structure through N‐H···N and N‐H···Cl hydrogen bonds (**Figure** **3**a).^[^ ^86^ ^]^ TAP displays the same number of hydrogen bonding donors, but the lack of a third hydrogen in the ring decreases the acceptor sites leading to a less extensive linear supramolecular network (Figure 3b), and AGA, owing to the methyl group displays a restricted supramolecular growth compared to melamine arising from steric effects (Figure 3c).^[^ ^87^ ^]^ The acyl and aryl groups also modify the solubility, the electron density in the heterocycle, and allow the synthesis of polymers through synthetic pathways beyond the commonly employed Schiff‐base reaction,^[^ ^44^ ^]^ such as Friedel‐Crafts reaction leveraging on the phenyl substituent in BGA.^[^ ^88^, ^89^ ^]^

While these organic molecules are substantially less explored than melamine for materials synthesis, they still show tremendous potential to address some of the drawbacks of melamine as building block, such as the high gap between the Highest Occupied Molecular Orbital (HOMO) and the Lowest Unoccupied Molecular Orbital (LUMO) which leads to wide bandgap semiconductor behavior in its condensates, its high condensation temperature, its sublimation or the lack of functional groups beyond the NH_2_ terminal units. In this article the aim is to provide a comprehensive overview of the state of the art in materials synthesis with N‐heterocyclic amines beyond melamine as well as the advantages and differences that each of these building blocks provides, screening through supramolecular interactions and with an special emphasis into covalent and carbon‐based materials, highlighting the materials properties and performance in fields such as sensing, photo‐chemistry, gas sorption and electrochemistry amongst others. Triazine cores comprise also one of the most utilized linkages for the synthesis of covalent organic frameworks (covalent triazine frameworks),^[^ ^90^ ^]^ given these are often made in situ via cyclotrimerization of nitriles rather than using melamine as precursors^[^ ^91^, ^92^, ^93^ ^]^ these materials won't be covered here and the reader is referred to previously published reviews on the topic.^[^ ^94^, ^95^, ^96^ ^]^

## 2 Benzoguanamine

**Figure 4:**
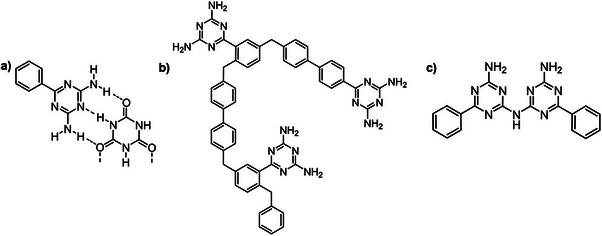
Representation of the hydrogen bonding interactions between BGA and cyanuric acid a),^[^ ^103^ ^]^ the structure of a Friedel‐Crafts polymer with 4,4‐bis(methoxymethane)biphenyl b),^[^ ^104^ ^]^ and the structure of a molecular dimer of BGA c).^[^ ^105^ ^]^

BGA has a phenyl group replacing one of the amine groups in melamine. The difference in the chemical structure results in marked differences on the electronic properties; while melamine displays a calculated HOMO LUMO gap of 6.86 eV corresponding to absorption at very short wavelengths arising exclusively from the π→π* transition,^[^ ^98^, ^99^ ^]^ BGA displays a shorter calculated gap of 5.37 eV due to the increased conjugation.^[^ ^100^ ^]^ BGA, like melamine, allows the formation of supramolecular materials, covalent organic polymers and oligomers derived from its self‐condensation (**Figure** **4**). However, due to the phenyl residue, beyond N─H···N complementary hydrogen bonds BGA can interact via π··· π, as well as C‐H··· π or metal···π interactions.^[^ ^101^, ^102^ ^]^

### 2.1 BGA‐Based Polymers

**Figure 5:**
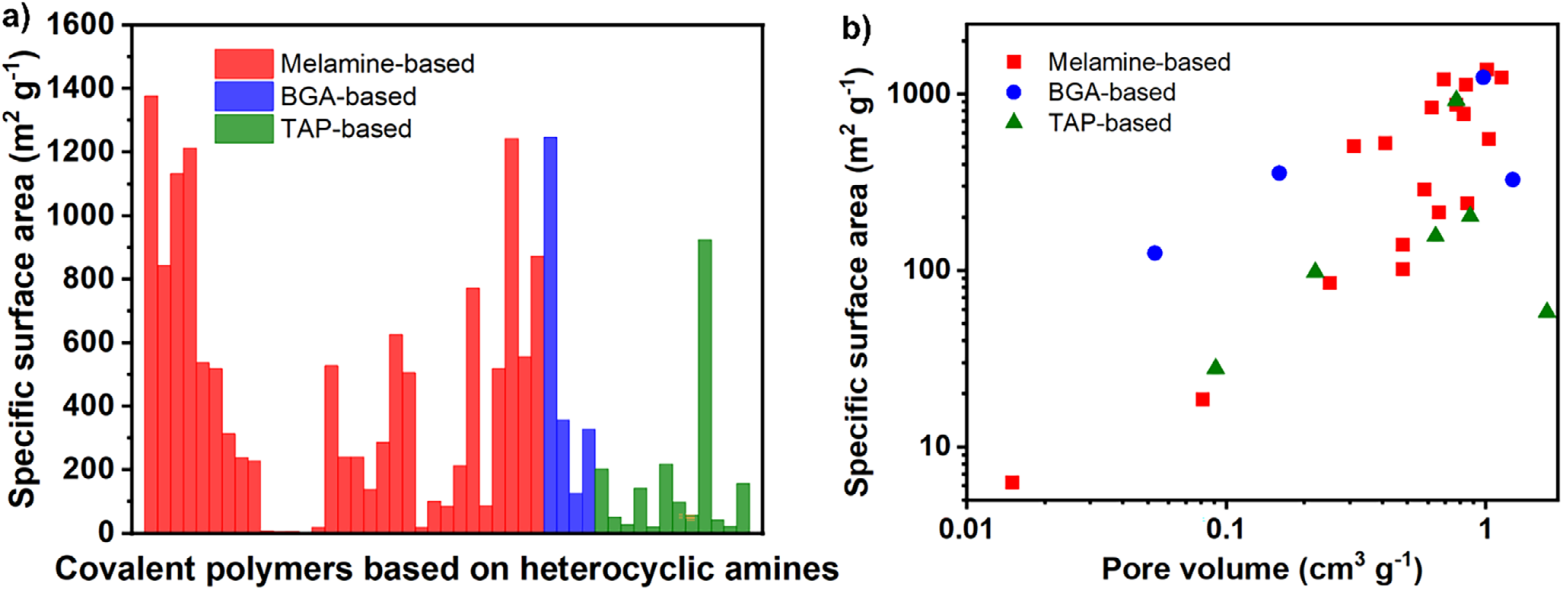
Specific surface area of covalent polymers synthesized with heterocyclic amines a) and logarithmic plot of the surface area – pore volume correlation b). Data obtained from Table 1.

BGA displays similar functional groups to perform Schiff‐base and condensation reactions and therefore it has been used as reinforcer in melamine‐based formaldehyde resins.^[^ ^106^, ^107^ ^]^ However, the number of polymers reported with BGA beyond resins is dramatically lower compared to melamine (**Figure** **5** and **Table** **1**). Ramasamy and co‐workers developed BGA‐based covalent organic polymers via reaction between BGA and terephthalaldehyde (1:2 mol ratio) in dimethylsulfoxide (DMSO) at 180 °C for 72 h under N_2_ atmosphere.^[^ ^108^ ^]^ During the reaction, an imine bond is formed, that subsequently reacts with an amine to form aminal bonds which were observed in the ─C─H transformed infrared spectroscopy (FTIR) vibration (at 2918 cm^−1^). The linkage of triazine and benzene cores with aminal bonds leads to the formation of polymer with a specific surface area of 125.7 m^2^ g^−1^. Owing to the intrinsic porosity, the polyaminal polymers were used as a template to prepare porous carbon materials through KOH‐activation and pyrolysis under N_2_ atmosphere at 700 °C, which exhibit CO_2_ uptakes of 2.89 mmol g^−1^. Moradi and co‐workers also employed BGA to synthesize a covalent organic framework linked via imine bonds rather than aminal bonds. In this work BGA reacted with a trialdehyde (2,4,6‐tris(4‐formylphenoxy)‐1,3,5‐triazine) in *n*‐butanol/*o*‐dichlorobenzene with acetic acid as catalyst. The resulting was a crystalline covalent organic framework with hexagonal pores of 27 Å diameter and a surface area of 328 m^2^ g^−1^.^[^ ^109^ ^]^ The combination of metal···π (via the phenyl group) and electrostatic interactions (via the triazine ring) led to a Hg^2+^ adsorption capacity of 1826 mg g^−1^, which, albeit not the highest,^[^ ^110^ ^]^ is a very competitive adsorption performance compared to other covalent frameworks and polymers.^[^ ^111^, ^112^ ^]^

**Table 1:** Literature survey on specific surface area and pore volume of covalent organic polymers synthesized with heterocyclic amines.

Heterocyclic amine	Co‐monomer	Surface area [m^2^ g^−1^]	Pore volume [cm^3^ g^−1^]	References
Melamine	Terephthalaldehyde	1377	1.01	[44]
Melamine	4,4’‐biphenyldicarboxaldehye	842	0.62	[44]
Melamine	Isophthalaldehyde	1133	0.84	[44]
Melamine	Tris(4‐formylphenyl)benzene	1213	0.69	[44]
Melamine	Terephthalaldehyde	537	‐	[45]
Melamine	Terephthalaldehyde + o‐phthalaldehyde	519	‐	[45]
Melamine	Terephthalaldehyde + o‐phthalaldehyde	314	‐	[45]
Melamine	Terephthalaldehyde + o‐phthalaldehyde	239	‐	[45]
Melamine	Terephthalaldehyde + o‐phthalaldehyde	228	‐	[45]
Melamine	Terephthalaldehyde + o‐phthalaldehyde	7	‐	[45]
Melamine	0‐phthalaldehyde	5	‐	[45]
Melamine	Terephthalaldehyde	6.3	0.015	[46]
Melamine	isophthalaldehyde	4.3	0.009	[46]
Melamine	O‐phthalaldehyde	18.7	0.081	[46]
Melamine	Terephthalaldehyde	528	0.41	[47]
Melamine	Terephthalaldehyde	239.6	‐	[49]
Melamine	Terephthalaldehyde	239.6	0.85	[50]
Melamine	4‐ethyl‐2,6‐diformylphenol	139.2	0.48	[50]
Melamine	3,5‐diformyl‐4‐hydroxybenzoic acid	287.4	0.58	[50]
Melamine	Terephthaldalehyde	626	‐	[60]
Melamine	2‐aminoterephthalic acid	507	0.31	[114]
Melamine	Phenantroline‐2,9‐dialdehyde	18.01	‐	[115]
Melamine	‐	102	0.48	[116]
Melamine	Benzophenone	85	0.25	[116]
Melamine	Aromatic diketone	213	0.66	[116]
Melamine	Aromatic triketone	772	0.82	[116]
Melamine	Isophthalaldehyde	86.1	‐	[117]
Melamine	Thieno[2,3‐b]thiophene‐2,5‐dicarboxaldehyde	519	‐	[118]
Melamine	Tetraphenyladamantane	1242	1.15	[119]
Melamine	Tris(4‐formylphenyl) amine	557	1.03	[120]
Melamine	1,3,5‐tris(4‐formylphenyl)‐benzene	873	0.77	[120]
BGA	4,4‐bis(methoxymethyl)biphenyl	1247	0.978	[113]
BGA	4,4‐bis(methoxymethyl)biphenyl	357	0.16	[88]
BGA	Terephthalaldehyde	125.7	0.053	[121]
BGA	2,4,6‐tris(4‐formylphenoxy)‐1,3,5‐triazine	328	1.27	[109]
TAP	Terephthalaldehyde	203	0.87	[122]
TAP	Isophthalaldehyde	51		[123]
TAP	1,4‐phenylene diisocyanate	27.95	0.091	[124]
TAP	1,4‐phenylene diisocyanate	142	‐	[125]
TAP	Aromatic triketone	21	‐	[116]
TAP	Terephthalaldehyde	218.8	‐	[126]
TAP	1,5‐diisocyanatenaphthalene	98	0.22	[127]
TAP	Cyanuric chloride	58.1	1.72	[128]
TAP	Tetraphenyladamantane	925	0.77	[119]
TAP	Terephthalaldehyde	43	‐	[129]
TAP	1,3,5‐triformylbenzene	21.7	‐	[129]
TAP	1,3,5‐triformylphloroglycinol	156.6	0.64	[130]

The phenyl group within BGA can also be leveraged to synthesize hypercross‐linked polymers via Friedel‐Crafts reaction in the presence of a metal chloride. Petit and co‐workers explored this concept via reaction of BGA with 4,4’‐bis(methoxymethyl)biphenyl in 1,2‐dichloroethane with iron(III) chloride.^[^ ^88^ ^]^ The synthesized polymers are built upon methylene bridges, and the introduction of triazine moieties increased not only the CO_2_ and water sorption capacities (compared to the reference polymers prepared with either benzene or aniline),^[^ ^113^ ^]^ but also the photocatalytic CO_2_ conversion due to a substantially higher electron – hole separation efficiency and electrostatic interaction between CO_2_ and the triazine ring.

### 2.2 BGA Self‐Condensates

**Figure 6:**
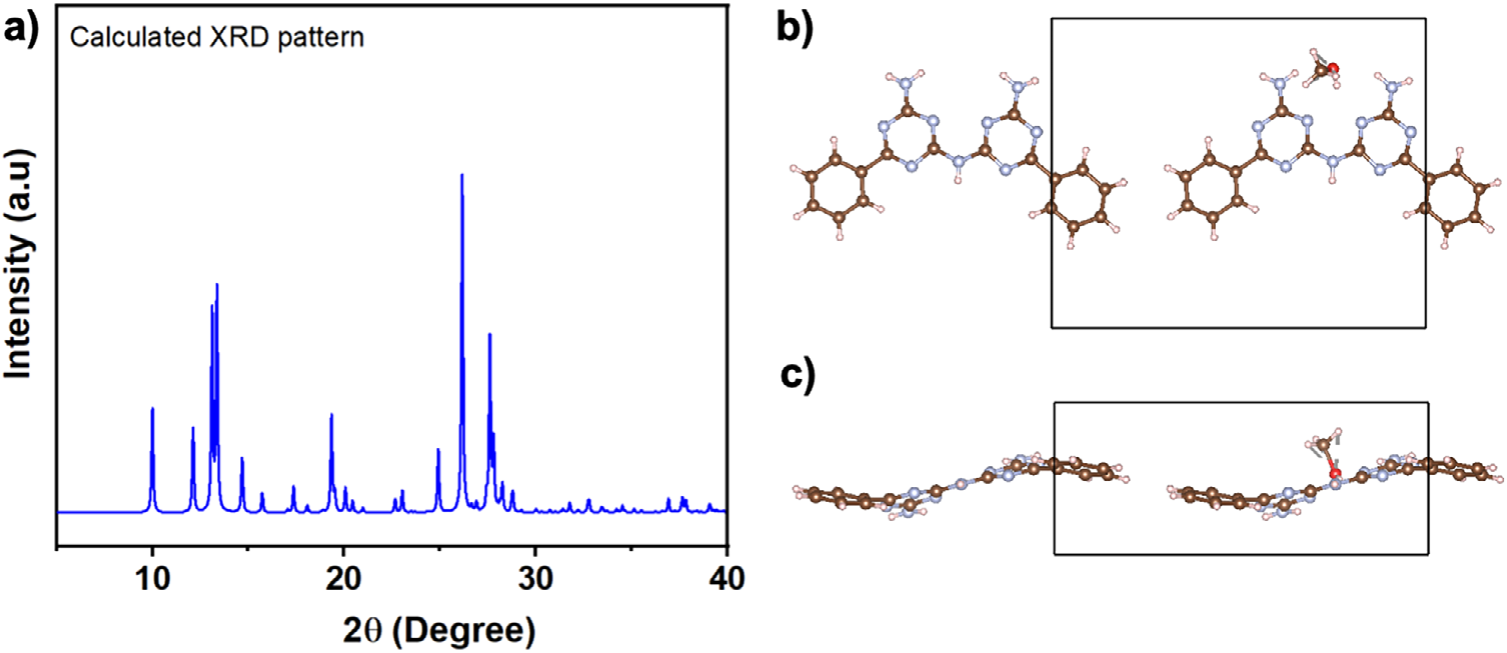
Calculated XRD patterns of BGA dimer a) and asymmetric unit of the BGA dimer crystallized in methanol along the c axis b) and a axis c). Reproduced with permission.^[^ ^105^ ^]^ Copyright 2021, ACS Publications. Hydrogen atoms are represented in pink, nitrogen atoms in blue, carbon atoms in brown, oxygen atoms in red and chlorine atoms in green. Structural visualization was carried out using the VESTA software.^[^ ^97^ ^]^

The self‐condensation of BGA has been recently studied owing to the strong fluorescent behavior of the reaction products, which was initially observed when BGA was used as a dopant in the synthesis of carbon nitride materials.^[^ ^131^ ^]^ Compared to melamine, BGA allows the synthesis of covalent frameworks at a lower condensation temperature (350 °C) due to the electron‐withdrawing nature of the phenyl substituent while also retaining part of the phenyl functional groups, enabling the framework functionalization.^[^ ^132^, ^133^ ^]^ This concept was initially proven by Sun et al. which synthesized modified BGA molecules via reaction of either 4‐chlorobenzonitrile, p‐tolunitrile or 4‐methoxybenzonitrile with dicyanamide under microwave irradiation and performed their thermal treatment at 350 °C under N_2_ atmosphere.^[^ ^134^ ^]^ The relatively low condensation temperature allowed the functional groups to be retained and therefore the electron‐donating or withdrawing nature of the groups could be correlated with the band structure and emission. The condensation of BGA at such temperatures, however, results in mixtures of the monomer and higher condensates which owing to its melting point can lead to the formation of supramolecular glasses.^[^ ^135^ ^]^ In this regard, Xu and co‐workers reported a solvent purification protocol which allowed the isolation of a BGA‐dimer and even the elucidation of its structure via single‐crystal X‐ray diffraction (XRD).^[^ ^105^ ^]^ In this work, after the thermal treatment of BGA at 370 °C, mass spectrometry revealed three majoritarian fragments with molecular weights of 188, 358, and 528 g mol^−1^, which correspond to the proton adducts of the monomer, dimer and trimer and which explains the presence of several emission peaks. Through consecutive solvent washing steps (with ethyl acetate and methanol), the molecular BGA dimer was isolated with a 92% purity and recrystalized in a tetrahydrofuran (THF) /methanol mixture. Single crystal studies confirmed that each BGA dimer displays intermolecular hydrogen bonding with a MeOH residue and neighbour molecules and π···π interactions with adjacent dimers (**Figure** **6**). Interestingly, the BGA dimer displayed aggregation‐induced emission and while in diluted THF the emission was negligible, the solid material achieved a 58% photoluminescence quantum yield, making an excellent latent fingerprint label.

However, Cheng et al. showed that even the unpurified reaction product of BGA condensation displayed a quantum yield of 30% in organic media in the presence of a surfactant, which allowed its application as cell fluorescent probe for cell imaging.^[^ ^136^ ^]^ The charge separation of such oligomeric reaction intermediates were studied by Wang et al. which confirmed via density functional theory calculations that the HOMO LUMO gap decreased with pyrolysis temperature and that the presence of long‐lived triplet states could be responsible for the distinct photoluminescent behavior.^[^ ^137^ ^]^

Further insights on the structure of the polymerized product were provided by Tian et al. who investigated the polymerization of BGA using chromatography and nuclear magnetic resonance (NMR). They observed the formation of dimers and trimers at lower temperatures (350 °C), followed by their decomposition into benzonitrile and cyanamide, which subsequently led to the formation of melem intermediates.^[^ ^138^ ^]^

### 2.3 BGA as Precursor for CNX Materials

Beyond its role in molecular and oligomeric architectures, BGA serves as a versatile precursor for the design of extended CNX materials via pyrolysis at high temperatures (> 400 °C). For instance, its triazine core and phenyl substituent offer a unique platform for tuning the electronic and structural properties of carbon nitride and boron carbon nitride frameworks.^[^ ^139^ ^]^ Shalom and co‐workers utilized a BGA‐containing supramolecular assemblies and crystals as reactant for the synthesis of polymeric carbon nitride with extended optical absorption, enhanced electronic conductivity and photo‐catalytic activity.^[^ ^103^, ^140^ ^]^ However, the main advantage of BGA over other conventional carbon nitride precursors is its melting point, which allowed the liquid‐based growth of homogeneous carbon nitride layers on electrodes,^[^ ^141^ ^]^ a system that has been explored with different metal catalysts,^[^ ^142^, ^143^ ^]^ and for different oxidation reactions in a photo‐electrochemical cell, owing to the *n*‐type character of carbon nitride.^[^ ^144^, ^145^, ^146^, ^147^ ^]^

**Figure 7:**
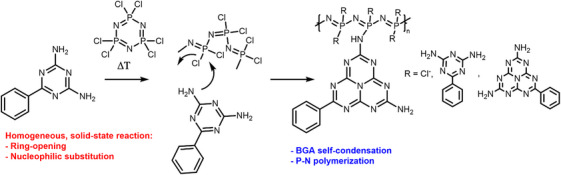
Schematic representation of the proposed reaction mechanism between BGA and phosphonitrilic chloride trimer at high temperatures in a molten‐state. Adapted from [148].

The relatively low melting point of BGA enables its use in liquid‐phase synthesis of carbon‐based frameworks, facilitating the formation of ternary CNX materials with a high degree of homogeneity. This approach ensures intimate mixing at the molecular level promoting the uniform incorporation of heteroatoms and therefore tuning the chemical and electronic structure. Zhang et al. demonstrated this concept highlighting the differences in reactivity between BGA, dicyanamide and melamine when reacting at various temperatures (450–850 °C) with phosphonitrilic chloride trimer ((PNCl_2_)_3_), a cyclic inorganic compound containing phosphorus and nitrogen atoms. The molten‐state transition of BGA at 260 °C allows its efficient and homogeneous liquid‐state reaction with the P‐N monomer, leading to its ring opening, the nucleophilic substitution of chlorine by the amine groups in BGA (**Figure** **7**) and the synthesis of a CN_x_P_y_ material with up to 32 wt.% phosphorus content.^[^ ^148^ ^]^ However, when using other C‐N molecules, given the absence of a clear molten phase, no reaction happened between the co‐monomers which led to a graphitic‐carbon nitride product with residual phosphorus content.

Additionally, the higher C/N ratio of BGA compared to melamine enables its use as precursor for conductive nitrogen‐doped carbon materials. This expands the applicability of BGA‐derived materials beyond photo‐electrocatalysis and fluorescent sensing to electrocatalysis, where nitrogen‐doped carbons are widely explored. In fact, metal‐containing nitrogen‐doped carbons, or metal‐nitrogen‐carbons (MNCs), are one of the most active electrocatalysts for both the electrochemical oxygen reduction reaction and the CO_2_ reduction reaction.^[^ ^149^, ^150^, ^151^ ^]^ They are typically synthesized via high‐temperature pyrolysis of carbon and nitrogen‐containing building blocks with a metallic salt.^[^ ^152^ ^]^ However, this process often leads to the formation of carbon‐coated nanoparticles due to metal‐catalysed graphitization.^[^ ^153^ ^]^

Rauf et al. employed the BGA‐containing supramolecular assembly earlier reported by Isida et al.^[^ ^103^ ^]^ along with FeCl_3_.6H_2_O and a commercial carbon material (KJ600 carbon black) to synthesize Fe nanoparticles encapsulated within nitrogen‐doped carbon nanotubes.^[^ ^154^ ^]^ The mixture was pyrolyzed at temperatures ranging from 700 to 900 °C, where BGA proved crucial to modulate the morphology, surface area and electrocatalytic performance in the alkaline oxygen reduction reaction.

## 3 Acetoguanamine

AGA displays a methyl group replacing one amino group on the triazine ring of melamine and maintains the same triazine core. While AGA is still capable to establish intermolecular hydrogen bonds, the presence of a ‐CH_3_ group decreases the number of hydrogen bond donors as well as the solubility in polar solvents. In fact, solubility data obtained by Yao and Bretti,^[^ ^155^, ^156^ ^]^ suggest that melamine displays a three order of magnitude higher solubility in pure water compared to that of AGA: 0.027 vs 1.26 × 10^−5^ mol L^−1^ at 298.15 K (solubility data for AGA in water was converted from the calculated molar fraction using Equation (1))

(1) $$C=x\frac{\rho }{M}$$

where C stands for molar concentration (mol L^−1^), *x* is the measured molar fraction of AGA in water at 298.15 K, ρ is the density of the solvent and M is the molar mass of the solvent.

**Figure 8:**
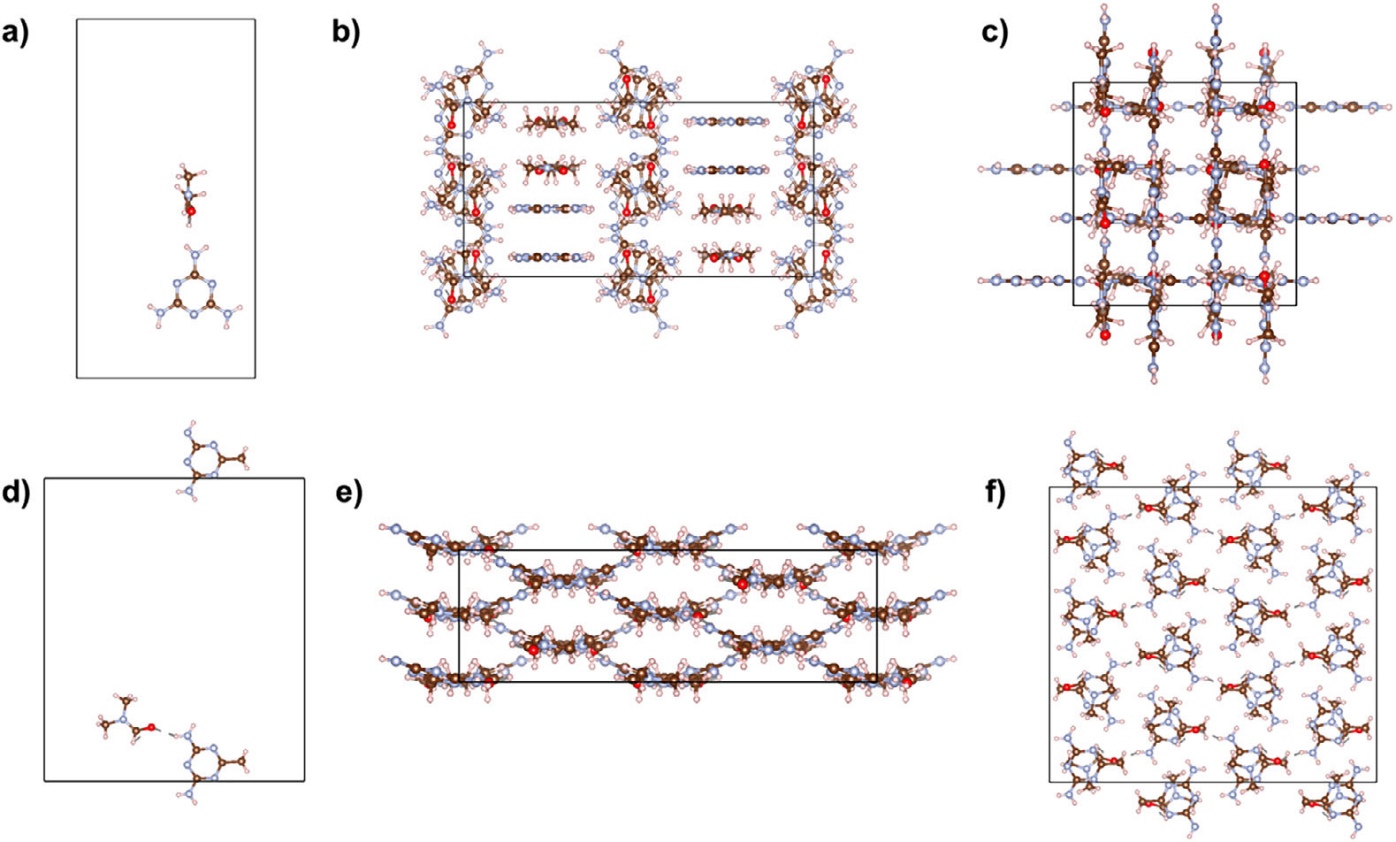
Asymmetric units of the melamine‐DMF a) and AGA‐DMF d) crystal structures as well as their packing structure along the a axis (b and e, respectively) and the c axis (c and f, respectively). Hydrogen atoms are represented in pink, nitrogen atoms in blue, carbon atoms in brown, oxygen atoms in red and chlorine atoms in green. Structural visualization was carried out using the VESTA software.^[^ ^97^ ^]^

While part of the large gap in the experimental solubility data for both monomers could be due to experimental variations (such as the determination method), the difference can be attributed to the structural differences. AGA displays less hydrogen bonding interactions with water molecules reducing its solubility. Additionally, the XRD patterns observed by Yao et al. of AGA‐H_2_O, display the same diffraction pattern than that of AGA, while Chen and co‐workers observed the formation of a crystalline hydrogen bonded framework of melamine in H_2_O.^[^ ^157^ ^]^ The higher solubility of AGA in Dimethylformamide (DMF) however resulted in a different crystalline pattern owing to the formation of a solvated crystal reported initially by Portalone et al,^[^ ^158^ ^]^ whose structure differs substantially from that of melamine crystallized in DMF (**Figure** **8**). While melamine establishes 9‐fold hydrogen bonds with six nearby molecules and one DMF, AGA forms ribbons stabilized by three N‐H···N and one N‐H···O hydrogen bonds with AGA and DMF, respectively, which is a similar structure to that observed in co‐crystals of AGA with various carboxylic acids.^[^ ^159^ ^]^

Further differences in reactivity can be observed in the Schiff‐base chemistry of AGA; melamine has been widely reported to form covalent microporous polymers with different aldehydes (Table 1), however, Erdem and co‐workers reported the synthesis of discrete Schiff bases based on AGA and different aldehydes, which were sequentially used as ligands to fabricate metal‐organic complexes.^[^ ^160^, ^161^ ^]^ This behavior can be attributed to the reduced nucleophilicity of the ‐NH_2_ groups in AGA and its lower molecular symmetry compared to melamine, both of which decrease the likelihood of forming extended networks.

Krishnan et al. later compared the AGA, BGA and melamine in the Schiff‐base reaction with hydroxynaphthaldehyde to prepare benzoxazine coatings. The utilization of AGA and BGA led to imine‐linked bifunctional monomers, while the utilization of melamine led to a trifunctional monomer (owing to the three amine groups). The benzoxazine was then synthesized via Mannich reaction with furfurylamine and paraformaldehyde. The higher functionality of melamine (establishing three imine linkages instead of two) led to the most resistant coating and a 41% char yield after treatment at 850 °C.^[^ ^162^ ^]^

### 3.1 AGA as Precursor for CN and BN Materials

Although there are a few reports of AGA being used to synthesize Schiff bases, cross‐linked hybrid materials,^[^ ^163^, ^164^ ^]^ or as an additive in electrochemical cells,^[^ ^165^ ^]^ in general it is the melamine analogue that has been less explored for materials synthesis. Wan et al. employed AGA as a modifier in the melamine‐cyanuric acid supramolecular framework to modulate the electronic structure and photocatalytic activity of the carbon nitrides obtained after pyrolysis under N_2_ atmosphere. The insertion of AGA in the supramolecular assembly led to the narrowing of the bandgap down to 1.78 eV when using equimolar amounts of melamine and AGA in the supramolecular assembly, and therefore a substantial enhancement of the visible light‐driven photocatalytic activity.^[^ ^166^ ^]^ However, recent reports have suggested that smaller doping amounts in a melamine‐AGA co‐crystal (5% in mol) and its utilization to synthesize carbon nitride result in a distinct fluorescent behavior in the solid state due to dramatic changes in the electron density distribution when inserting a ‐CH_3_ group in the melem moiety.^[^ ^167^ ^]^ In this case, the electron density near the hydrogen atoms is increased compared to the case of ‐NH_2_ groups, which alters the HOMO LUMO distribution.

The self‐condensation of AGA to fabricate covalent materials has also been studied. Li et al. compared the products from the low‐temperature thermal treatment of AGA and TAP; them both being isomers with a different spatial arrangement.^[^ ^168^ ^]^ The different location of the carbon atom (within the ring in the case of TAP, and as a methyl substituent in the case of AGA) dramatically impacted the stability, chemical composition and electronic structure of its condensates. Like BGA, both AGA and TAP lead to the formation of narrow bandgap semiconductor materials (1.92 and 1.59 eV, respectively) at a low condensation temperature (300 °C), therefore maintaining the ‐NH_2_ and ‐CH_3_ functional groups, which enhances the processability in liquid media. Similar findings were recently reported by Zhang and co‐workers, who corroborated the low pyrolysis temperature required by AGA to enable ring‐opening reaction and polymerization.^[^ ^85^ ^]^

**Figure 9:**
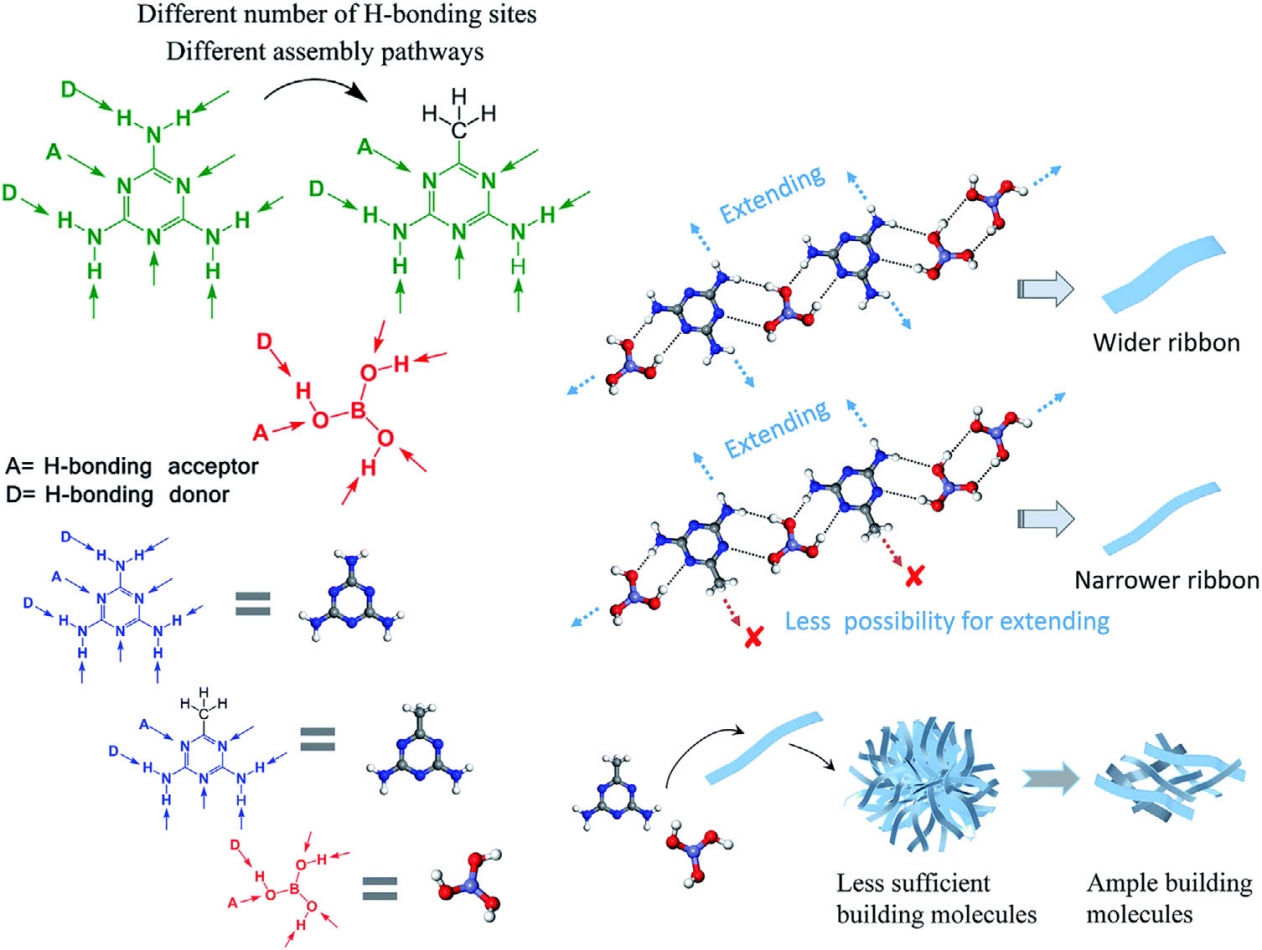
Representation of the hydrogen bonding interaction between melamine and AGA with boric acid and the implications on boron nitride morphology. Reproduced with permission.^[^ ^170^ ^]^ Copyright 2020, Royal Society of Chemistry.

The differences between melamine and AGA have also been leveraged to construct boron nitride‐based superstructures via pyrolysis of melamine‐boric acid and AGA‐boric acid aerogels.^[^ ^169^ ^]^ Pan et al. studied the impact of the low dimensionality from the AGA‐boric acid aerogels on the properties of the final boron nitride structures (**Figure** **9**);^[^ ^170^ ^]^ the lack of hydrogen bonds established through the ‐CH_3_ group in AGA influenced the assembly pathways leading to very narrow nanoribbons compared to those formed with the melamine‐boric acid crystal and interestingly, to a highly hydrophobic behavior.

## 4 2,4,6‐Triaminopyrimidine

**Figure 10:**
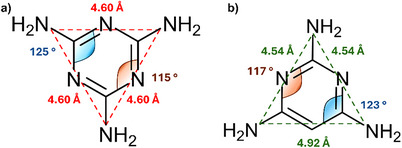
N‐C‐N and C‐N‐C angles as well as amine‐amine distances of melamine a) and TAP b). Adapted from [119].

**TAP** displays a **pyrimidine (1,3‐diazine) core,** which differs from melamine (1,3,5‐triazine core) by having only **two nitrogen atoms in the ring**. TAP has been widely studied in supramolecular chemistry for nucleobase recognition, as it shares the heterocycle with canonical and noncanonical nucleobases such as uracil and its derivatives and it can interact via hydrogen bonding with their oxygen functional groups.^[^ ^171^ ^]^ Additionally, similar to the melamine‐cyanuric acid system,^[^ ^172^ ^]^ the supramolecular assembly of TAP with either cyanuric or barbituric acid has led to a wide variety of supramolecular polymers,^[^ ^173^, ^174^, ^175^, ^176^ ^]^ which have also been leveraged in the field of materials science.^[^ ^177^, ^178^, ^179^ ^]^ However, TAP has also been shown to perform hydrogen bonding with the oxygen functional groups within polymers such as polyethylene oxide methacrylate, modulating the free volume within polymer membranes and boosting CO_2_ separation selectivity.^[^ ^180^ ^]^ While TAP behaves similarly to melamine in supramolecular and condensation chemistry, the lack of a third nitrogen atom in the ring differentiates its reactivity and geometry. For instance, the C─C bond (1.39 Å) is longer than the C‐N bond (1.33 Å), which means that the inner N‐C‐C and C‐N‐C angles in TAP (123 and 117° respectively) differ from the N‐C‐N and C‐N‐C of melamine (125 and 115°), and therefore the side length between amino groups that straddle the extra carbon atom is 4.92 Å instead of 4.60 Å (**Figure** **10**).^[^ ^119^ ^]^ The presence of an additional C─H within the ring influences also the electronic structure and basicity. Both melamine and TAP can be protonated in the heterocyclic nitrogen atoms,^[^ ^181^, ^182^, ^183^ ^]^ but TAP exhibits a higher basicity due to the C‐H group, which reduces the electron withdrawal from the ring and results in a first pKa of 6.8 compared to 5.0 for melamine.^[^ ^184^ ^]^ This same electronic effect decreases the basicity of the ‐NH_2_ groups in TAP, making them less nucleophilic and less likely to attack an imine bond formed in a Schiff‐base synthesis.^[^ ^119^ ^]^

### 4.1 TAP‐Based Polymers

**Figure 11:**
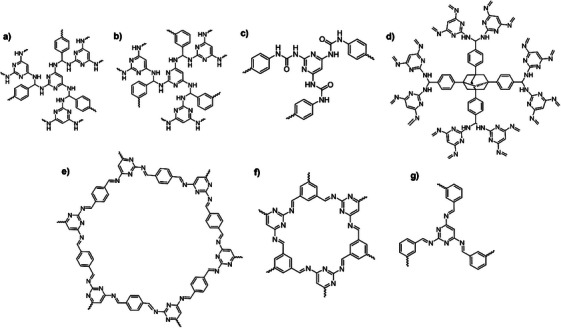
Covalent organic polymers and frameworks based on the TAP building block comprising aminal a,b,d), urea c) and imine e,f,g) linkages. Reproduced with permission.^[^ ^119^, ^122^, ^123^, ^125^, ^129^ ^]^ Copyright 2014, ACS Publications. Copyright 2018, Wiley. Copyright 2023, MDPI. Copyright 2014, ACS Publications. Copyright 2019, Wiley.

The structural similarities between TAP and melamine have led to its study as building block to construct Schiff‐base polymers. Melamine‐based polymers typically attain higher microporosity and specific surface area owing to melamine symmetry, which promotes the formation of highly cross‐linked frameworks 3D frameworks (Figure 5 and Table 1). In contrast, TAP, with its lower symmetry, is more prone to forming 2D or less rigid networks. Nevertheless, TAP can also offer advantages, particularly in applications where larger pore size or less rigid frameworks are desirable. Wang et al. reported over a decade ago the synthesis of a polyaminal‐linked polymer via reaction between TAP and Tetraphenyladamantane and its utilization for CO_2_ capture. They observed that, unlike melamine, the NH_2_ groups of TAP display a lower basicity and therefore the polymer comprised both aminal and imine linkages (**Figure** **11**d).^[^ ^119^ ^]^ This fact strongly impacted the pore size and specific surface area; the melamine‐based polyaminal displayed 1242 m^2^ g^−1^, with 1.15 cm^3^ g^−1^ pore volume and a pore size ranging 0.39‐0.71 nm, while the TAP‐based imine/aminal‐linked displayed 925 m^2^ g^−1^, with 0.77 cm^3^ g^−1^ and a pore size ranging 0.57–1.36 nm. However, the bigger pore size in the TAP‐based covalent polymer allowed for a higher adsorption capacity of molecules such as benzene or cyclohexane even in the low‐pressure region. Shortly after, Bhunia et al performed the condensation between TAP and 1,4‐phenylene diisocyanate to form a porous polymer linked through urea moieties; a porous polyurea network. Unlike a conventional Schiff‐base polymer linked via imine or aminal bonds, the TAP‐based polyurea porous network displayed hydrogen bond donor‐donor‐acceptor arrays, which made it suitable to interact with nucleobases or drugs, therefore expanding the applicability of such polymers in the field of sensing or drug‐delivery carrier.^[^ ^125^ ^]^

Following Wang's report, several Schiff‐base polymers have been reported with TAP and different aldehydes (Table 1 and Figure 11),^[^ ^185^ ^]^ albeit discrepancies can be found as for which linkage prevails, whether the imine or the aminal bond. Pitchumani and co‐workers synthesized a porous organic polymer via Schiff‐base reaction between TAP and terephthalaldehye in DMF in a pressurized autoclave at 180 °C for 72 h.^[^ ^122^ ^]^ Via ^15^N solid state NMR just two different chemical environments for nitrogen were observed, which were correlated to the pyrimidine ring and the aminal bonds. With the same synthetic protocol, albeit a reaction temperature of 120 °C and reaction time of 48 h in a pressurized pyrex tube, Mandal et al. did actually observe ^13^C NMR signals corresponding to both aminal and imine linkages, suggesting that not all the imine moieties were converted into aminal units.^[^ ^126^ ^]^ Pinto and co‐workers observed as well the presence of both imine and aminal linkages in the polymer product of the reaction between TAP and isophthalaldehyde in a Schlenk flask in DMSO at 160 °C for 72 h,^[^ ^123^ ^]^ and Chen et. al. reported a mechanochemically‐synthesized imine‐linked covalent organic framework via reaction of TAP and terephthalaldehyde as well as TAP and 1,3,5‐triformylbenzene.^[^ ^129^ ^]^ The differences in reactivity and therefore the observed linkages could be due to the synthesis set up and protocol.^[^ ^186^ ^]^ The utilization of a pressurized autoclave can lead to changes in the thermophysical properties of the solvent according to the temperature and pressure,^[^ ^187^ ^]^ which can affect the reactivity of the intermediates and shift the reaction equilibrium toward the aminal bond formation. In the particular case of TAP‐based polymers reported in the literature, given the imine bond is reversible, at high temperatures under pressurized environment and prolonged reaction time, a full aminal conversion could be more likely due to factors such as: the increased solubility of the intermediates, faster reaction rates, the lower activation barriers or the enhanced imine hydrolysis (if trace water is present). While reports suggest also the synthesis of TAP‐based polymers via electrochemical polymerization,^[^ ^188^ ^]^ similar to the one reported by Klinke and co‐workers for the case of melamine‐based poly‐triazine imides,^[^ ^189^ ^]^ nowadays there is a lack of structural characterization of such TAP‐based polymers which hinders the elucidation of its structure.

The hierarchical porosity and hydrogen bonding donor and acceptor sites within TAP‐based polymers has led to their application in fields such as heavy metal removal,^[^ ^124^, ^190^ ^]^ conversion of epoxides to cyclic carbonates,^[^ ^126^ ^]^ and as organocatalyst for hydrogen bond donating reactions.^[^ ^127^, ^128^ ^]^ However, the wide variety of linkages, as well as aldehydes employed in the synthesis of such polymers and Schiff‐bases allows the fine tuning of the properties for the application in fields beyond adsorption or catalysis (Figure 11). For instance, in electrochemical devices; Nie et al. used TAP as an additive in melamine‐perylene diimide polymers to tune the carrier transport and photo‐electrocatalytic activity,^[^ ^191^ ^]^ and Zhang and co‐workers correlated the pore size and interlayer distance of two different imine‐linked TAP‐based covalent organic frameworks to the Li^+^ storage capacity in Li‐ion batteries.^[^ ^129^ ^]^ But also in polymer science. Chen et. al. reported a TAP‐containing urea‐bonded shape memory polymer via reaction between isophorone diisocyanate, 1,4‐Bis(3‐aminopropoxy) butane, and TAP which aimed to mimic the muscle fiber. The hydrogen bonding donor and acceptor sites within the urea linkages as well as the covalent cross‐linking led to excellent mechanical properties and environmental stability, being able to hold up to 25000 times its weight.^[^ ^192^ ^]^ Guo et. al. designed a TAP‐based catechol‐containing Schiff‐base which was linked to a polyfluoroether to design a high‐performance elastomer that displayed high flexibility even under liquid nitrogen.^[^ ^193^ ^]^

### 4.2 TAP as Precursor for Carbon Materials

Unlike melamine, which yields C_3_N_4_‐like materials after thermal treatment under inert atmosphere, TAP owing to its higher C/N ratio results in conductive nitrogen‐doped carbon frameworks after pyrolysis at high temperatures. The high conductivity and the robustness of such carbon frameworks can be leveraged in the field of electrocatalysis, where enough electronic conductivity as well as high number of accessible active sites are desired. In this regard, while carbon nitride‐based materials derived from melamine can provide a suitable platform for the design of active sites (such as single metal atoms coordinated in the nitrogen moieties),^[^ ^75^, ^194^ ^]^ the lack of electronic conductivity hinders their applicability in electrochemical reactions such as the oxygen or CO_2_ reduction. For such reasons, as well as the structural similarity to melamine, TAP has been leveraged also to synthesize CN‐based semiconductors at relatively low pyrolysis temperatures,^[^ ^168^, ^195^ ^]^ and to increase the carbon content and decrease the optical bandgap of carbon nitrides.^[^ ^196^, ^197^, ^198^ ^]^ Although it has also been explored as dopant to induce nitrogen functionalities in carbon materials.^[^ ^199^ ^]^

**Figure 12:**
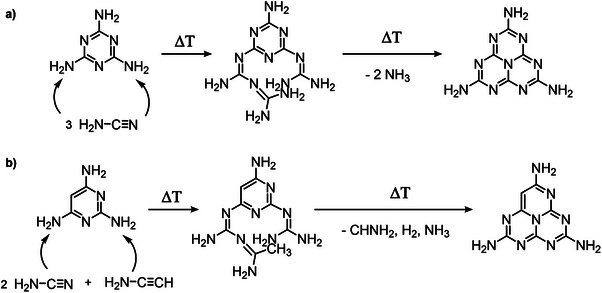
Suggested pathways for the formation of melem from melamine self‐condensation a), and for the formation of carbon‐rich melem‐like building blocks from TAP self‐condensation. Adapted from [66, 200].

Nevertheless, TAP has recently attracted attention as a sole carbon and nitrogen‐based precursors to fabricate conductive carbons. Titirici and co‐workers recently studied the self‐condensation pathway of TAP via thermogravimetry coupled to mass spectrometry and based on the observed fragments hypothesized that, similar to melamine, which breaks down in three cyanamine fragments,^[^ ^66^ ^]^ TAP breaks down in two cyanamide and one ethynamine fragments which attack unreacted TAP molecules leading to carbon‐rich, melem‐like reaction intermediates (**Figure** **12**). To leverage TAP in electrochemical applications, however, templating agents are needed to induce micro and mesopore volume due to the melting point of TAP (at 235 °C) which otherwise leads to compact non‐porous solids. Zhang and coworkers reported back in 2017 the utilization of a supramolecular assembly comprising TAP and barbituric acid as precursor to synthesize iron‐nitrogen‐carbon (FeNC) oxygen reduction electrocatalysts with a surface area of 232 m^2^ g^−1^.^[^ ^178^ ^]^ The pyrolysis of carbon and nitrogen precursors (in this case the TAP‐barbituric acid supramolecular assembly) with Fe species, however, leads to different Fe phases such as carbides, oxides, or metallic Fe which can catalyze the formation of graphitic shells. Titirici and co‐workers overcame the formation of Fe‐based aggregates by using TAP as precursor in a decoupled synthetic protocol comprising a Lewis‐acid mediated pyrolysis at high temperatures and a cation exchange protocol,^[^ ^200^ ^]^ previously reported by Mehmood et al.^[^ ^201^, ^202^ ^]^ The pyrolysis of TAP in the presence of a Lewis acid (such as MgCl_2_.6H_2_O) leads to a highly porousnitrogen‐dopedd carbon material (>3500 m^2^ g^−1^),^[^ ^203^, ^204^ ^]^ which allows a high accessibility of the active sites to an electrochemical media, a crucial parameter to enhance electrocatalytic activity. Therefore, the coordination of metallic ions such as Fe or Ni, in such frameworks has led to the fabrication of some of the most active catalysts for the oxygen reduction reaction (both in acidic and alkaline environments),^[^ ^204^, ^205^ ^]^ hydrogen fuel cells,^[^ ^206^ ^]^ and the electrochemical CO_2_ reduction to CO.^[^ ^207^ ^]^ Furthermore, the reliability of the synthetic protocol has allowed the coordination of alternative metallic centers such as Co and Cr, and therefore expanded the applicability to other electrochemical reactions.^[^ ^208^ ^]^

## 5 Conclusion and Outlook

While melamine‐based materials such as supramolecular assemblies or covalent organic polymers have been extensively studied during the last decades, analogues such as BGA, AGA or TAP offer diverse functionalities and reactivities that can be harnessed to engineer novel covalent organic materials, polymeric semiconductors or conductive carbon‐based frameworks. This review has highlighted the implications of employing N‐heterocyclic amines structurally related to melamine for materials synthesis, including their effect on condensation behavior, morphology, porosity and performance across various application. For instance, BGA can form homogeneous polymeric films owing to its melting point, and therefore, unlike melamine, does not require vapor‐based techniques for photo‐electrodes fabrication. It further enables photoactive polymers via Friedel Crafts reactivity for CO_2_ capture and conversion, inaccessible to melamine, and forms highly emissive oligomeric building blocks via self‐condensation for opto‐electronics. AGA, through its methyl substituent, alters the dimensionality of supramolecular assemblies compared to melamine's 3D growth, thereby influencing the resulting material after thermal treatment. TAP can form imine‐linked polymers owing to the lower nucleophilicity of its amine groups, which can lead to 2D layered polymers, contrasting with melamine's 3D porous organic polymers. Furthermore, TAP yields conductive, carbon‐based electroactive frameworks after high‐temperature pyrolysis suitable for electrochemical devices, unlike melamine, which leads to semiconductive polymers. Collectively, these contrasts highlight not only the chemical versatility of melamine analogues, but also their potential for breakthrough applications in energy conversion, optoelectronics, and advanced functional materials. With the aim of advancing the field, we outline several future directions below:

- Much can be learned from the mature field of melamine‐derived materials, particularly in guiding the design of Schiff‐base polymers. The use of alternative N‐heterocyclic amines in such reactions remains in its infancy. In this regard, the large library of available aldehydes provides a convenient means to modulate the framework's band structure, pore architecture, and dimensionality. Furthermore, the incorporation of redox‐ or light‐active molecules such as porphyrins or phthalocyanines could enhance their performance in photo and electrochemical applications, as already demonstrated with covalent organic frameworks and metal organic frameworks.^[^ ^209^, ^210^, ^211^, ^212^ ^]^ Additionally, BGA‐based Friedel‐Crafts polymerization routes remain virtually unstudied and could yield robust, C─C bonded porous frameworks with novel pore architectures and energy band positions.
- While certain progress has been achieved with Schiff‐base polymers, the structural identity of the condensation products (whether imines or aminals) is often ambiguous, particularly when novel N‐heterocycles are employed. Contradictory reports in the literature highlight the need for systematic studies on how monomer structure, solvent, and reaction conditions influence the polymer backbone. Understanding these structure‐activity relationships is essential to rationally design frameworks with predictable properties and topologies.
- Currently, TAP is the main N‐heterocycle employed to synthesize conductive carbon‐based materials from all the melamine analogues. Broadening this scope, not just to BGA or AGA, but also to their condensates and polymers, especially in conjunction with soft and hard templating agents,^[^ ^213^ ^]^ could offer new pathways to carbon materials with highly tunable porosity, surface chemistry, and catalytic performance.
- The formation of self‐condensed frameworks from N‐heterocyclic amines under thermal treatment remains poorly understood. These processes typically involve complex mixtures of monomers, dimers, and higher molecular weight oligomers, making it difficult to isolate and characterize pure products. In situ characterization techniques such as FTIR coupled to thermogravimetry, thermogravimetry‐mass spectrometry or in situ microscopy are essential to elucidate intermediate species and reaction mechanisms during thermal polymerization. Moreover, the development of straightforward purification protocols is critical to obtain well‐defined materials. In this regard, the utilization of molten salt media, widely studied for crystalline, condensed carbon nitride materials^[^ ^214^, ^215^, ^216^ ^]^ may pave the way toward the elucidation of the crystal structure of such condensates via X‐ray diffraction techniques.^[^ ^217^ ^]^
- As sustainability becomes a central concern in materials synthesis, low‐temperature, low‐waste, and solvent‐free approaches such as ball milling or water‐based synthesis have gained attention.^[^ ^218^, ^219^, ^220^ ^]^ These greener protocols are especially relevant for synthesizing both polymeric and carbon‐based materials from heterocyclic amines. Additionally, AI‐guided molecular design and automation platforms offer new opportunities to accelerate the discovery of optimal monomer combinations, reaction conditions, and performance‐structure relationships, enabling the rational design of next‐generation functional materials.^[^ ^221^, ^222^, ^223^ ^]^

In summary, the application of N‐heterocyclic amines opens new horizons for designing functional organic and carbon‐based materials. By integrating lessons from melamine chemistry with modern tools in synthesis, characterization, and computation, the full potential of these versatile building blocks can be achieved, enabling innovations in CO_2_ capture, water cleaning, photo‐electrocatalysis and energy storage.

## Conflict of Interest

The authors declare no conflict of interest.
